# Starch Bandage in Recent Fracture—Bellevue Hospital

**Published:** 1862-01

**Authors:** 


### STARCH BANDAGE IN RECENT FRACTURE--BELLEVUE HOSPITAL.

With few exceptions authors limit the immovable apparatus to those cases in which the inflammation and swelling following the injury have entirely subsided, and for the best of reasons. If applied as originally recommended, the dressing being perfectly unyielding, if subsequent swelling occurred at the seat of injury the most serious consequences might follow. The compression of the veins would readily become so great as to interrupt the return circulation so far as to produce gangrene of the extremity. Instances of this kind in which, after the dressings were applied, the swelling increased, and mortification occurred, early led to the strict rule of practice, never to apply the immovable apparatus until all danger from the subsequent swelling of the limb had clearly passed. The rule is a just one, and should never be departed from when the dressings applied in the manner first recommended.

There is a manifest advantage, however, in applying permanent dressings to simple fractures immediately after their occurrence. There is, then, no shortening of the limb to be overcome by subsequent traction, and no painful spasms of the muscles excited by the irritation of the fractured bone.—If the displaced fragments are placed and retained in perfect apposition, during the quiescent period that intervenes previous to the commencement of the reparative process, there will be less liability to swelling and subsequent inflammation. Besides, in private practice patients and friends are never satisfied unless the fracture "is set" immediately, the mere manipulations by which the fragments are opposed being with them the most important part of the whole treatment.

Admitted that the starch apparatus is well adapted to old fractures, is it possible to render it serviceable as a primary and yet permanent dressing? This question has now been definitely settled affirmatively. By first applying a thick layer of cotton wadding to the limb, as recommended by Burggraeve, of Ghent, adapting it nicely to all the irregularities of the parts, the starch apparatus may be at once applied in simple fractures with the happiest results. The cotton should completely énvelop the whole limb, and the first roller be placed over it. This should be applied firmly, and the application of starch should be first made to this bandage. The cotton is so elastic as to perfectly protect the superficial vessels from undue compression, even though swelling should follow. But the contrary effect is generally produced. Before the dressing is completed, the patient remarks that his limb feels pleasantly cool, and never that the dressing is too tight in the vicinity of the fracture. The result of this application of the starch is a rapid reduction of the swelling; thus rendering it the best local application that can be made.

We have recently seen the starch apparatus applied in this manner in Bellevue Hospital, to recent fractures of the leg, thigh, and arm, without the slightest inconvenience, but with immediate relief to swelling, and those painful startings and other symptoms attendant upon the exposure of the limb for several days without dressing. In fractures of the thigh, the patient is able to leave his bed as soon as the dressings are dry, generally about the third or fourth day after this application. Of course no weight is to be borne upon it. In fractures of the arm, where non-union is so common, the starch apparatus may be applied at once, and all dangers of such results be obviated. To country practitioners this dressing offers great advantages. The limb is at once firmly and securely put in a permanent dressing, without the slightest chance of displacement or other complication. The method of applying the immovable dressings is well illustrated in Erichsen's Surgery.—*American Medical Times.*

